# CXCL8 histone H3 acetylation is dysfunctional in airway smooth muscle in asthma: regulation by BET

**DOI:** 10.1152/ajplung.00021.2015

**Published:** 2015-02-20

**Authors:** Rachel L. Clifford, Jamie K. Patel, Alison E. John, Amanda L. Tatler, Lisa Mazengarb, Christopher E. Brightling, Alan J. Knox

**Affiliations:** ^1^Department of Respiratory Medicine and Nottingham Respiratory Research Unit, University of Nottingham, Nottingham, United Kingdom; and; ^2^Institute for Lung Health, Department of Infection, Immunity and Inflammation, University of Leicester, Leicester, United Kingdom

**Keywords:** airway smooth muscle, asthma, histone acetylation, bromodomain, BET protein, CXCl8

## Abstract

Asthma is characterized by airway inflammation and remodeling and CXCL8 is a CXC chemokine that drives steroid-resistant neutrophilic airway inflammation. We have shown that airway smooth muscle (ASM) cells isolated from asthmatic individuals secrete more CXCL8 than cells from nonasthmatic individuals. Here we investigated chromatin modifications at the CXCL8 promoter in ASM cells from nonasthmatic and asthmatic donors to further understand how CXCL8 is dysregulated in asthma. ASM cells from asthmatic donors had increased histone H3 acetylation, specifically histone H3K18 acetylation, and increased binding of histone acetyltransferase p300 compared with nonasthmatic donors but no differences in CXCL8 DNA methylation. The acetylation reader proteins Brd3 and Brd4 were bound to the CXCL8 promoter and Brd inhibitors inhibited CXCL8 secretion from ASM cells by disrupting Brd4 and RNA polymerase II binding to the CXCL8 promoter. Our results show a novel dysregulation of CXCL8 transcriptional regulation in asthma characterized by a promoter complex that is abnormal in ASM cells isolated from asthmatic donors and can be modulated by Brd inhibitors. Brd inhibitors may provide a new therapeutic strategy for steroid-resistant inflammation.

asthma is a chronic disease characterized by inflammation and remodeling of the airways. Clinical manifestations include repeated episodes of shortness of breath and wheezing, recurrent cough, and excess mucus production. Airway smooth muscle (ASM) cells are a primary contributor to asthma pathogenesis. Traditionally considered as purely a contractile cell, ASM cells contribute to airway inflammation and remodeling through the production of lipid mediators, chemokines (29), cytokines (47), growth factors (47), matrix metalloproteinases (17), and proangiogenic factors (11).

CXCL8 is a chemokine that mediates its effects via two G protein-coupled receptors, CXCR1 and CXCR2. It is implicated in the pathogenesis of a variety of inflammatory and malignant diseases including inflammatory bowel disease (24), rheumatoid arthritis (22), non-small cell lung cancer (53), and asthma. In asthma, CXCL8 is a highly potent neutrophil chemoattractant causing neutrophil recruitment into the airways (50). Levels of CXCL8 are 19 times higher in tracheal aspirates from asthmatic subjects compared with nonasthmatic subjects, and levels correlate with neutrophil number (42). Furthermore, asthmatic individuals have higher sputum, plasma, and exhaled breath condensate CXCL8 concentrations compared with healthy controls (12, 46). A higher proportion of airway epithelial cells express CXCL8 in asthmatic compared with nonasthmatic individuals (45), and we have shown that ASM cells isolated from asthmatic individuals express more CXCL8 protein and mRNA than those isolated from nonasthmatic controls (29). Identifying new mechanisms of CXCL8 modulation may therefore provide targets for treating severe asthma and other inflammatory diseases.

We showed previously that CXCL8 hypersecretion from ASM cells isolated from asthmatic donors was associated with increased binding of transcription factors and RNA polymerase II to the “asthmatic” CXCL8 promoter, but without an associated increase in transcription factor levels (29). Here we hypothesized that epigenetic modifications of the DNA and chromatin environment may underlie dysregulated CXCL8 transcription, specifically changes in either histone modifications or DNA methylation.

Histone acetyltransferases (HATs) catalyze the addition of acetyl groups to lysine residues present in core histone proteins, whereas histone deacetylases remove acetyl groups (31). Acetylated lysine residues are recognized by bromodomains, which exist in 46 diverse nuclear and cytoplasmic proteins including the bromodomain and extraterminal (BET) proteins (20). The mammalian BET protein group includes Brd2, Brd3, and Brd4. Inhibitors of BET proteins selectively interfere with gene expression programs that mediate cell growth, apoptosis, and inflammatory responses (14, 15, 40).

A further degree of complexity is added to epigenetic transcriptional regulation by DNA methylation, a reversible modification of cytosine residues mainly in which a cytosine is followed directly by a guanine (CpG sites) (52). CpG methylation regulates gene expression via complex mechanisms including via direct inhibition of transcription factor binding and recruitment of methyl-binding domain containing chromatin-remodeling complexes.

Here we assessed chromatin modifications and DNA methylation levels at the CXCL8 promoter in ASM cells isolated from individuals with and without asthma and explored the potential for BET protein inhibitors as modulators of aberrant CXCL8 expression. We found that histone H3 acetylation, specifically histone H3 lysine 18 acetylation (H3K18Ac), was increased at the CXCL8 promoter in ASM cells from asthmatic patients. Increased H3K18Ac was associated with increased recruitment of the histone acetyltransferase p300. In contrast, DNA methylation levels were similar in ASM cells from asthmatic and nonasthmatic donors. Finally, we found that CXCL8 expression can be modulated via BET protein inhibition of BRD4 and RNA polymerase II association with the CXCL8 promoter.

## MATERIALS AND METHODS

### 

#### Cell culture.

Primary cultures of human ASM (HASM) cells were isolated from bronchial biopsies and large airway tissue from subjects undergoing surgery at Glenfield Hospital, Leicester, UK and cultured as previously described (11, 29). The isolation and culture techniques were identical for asthmatic and nonasthmatic donors. Asthmatic subjects and nonasthmatic controls from which cells were isolated were recruited from Leicester, UK. Subjects with asthma had a consistent history and objective evidence of asthma, as indicated by one or more of the following: *1*) methacholine airway hyperresponsiveness [PC_20_ forced expiratory volume in 1 s (FEV_1_) <8 mg/ml]; *2*) >15% improvement in FEV_1_ 15 min after administration of 200 μg of inhaled salbutamol, or *3*) >20% of maximum within-day amplitude from twice daily peak expiratory flow measurements over 14 days. Severity of asthma was defined by Global Initiative for Asthma (GINA) guidelines (33). The study was approved by the Leicestershire Research Ethics Committees and all patients gave their written, informed consent (30). Four nonasthmatic individuals were nonsmokers and two were ex-smokers. Four of the asthmatic individuals were nonsmokers and three were ex-smokers. To maintain statistical power we did not distinguish between non- and ex-smokers in our observations. Cells were cultured in DMEM (Sigma) containing 10% fetal bovine serum (GIBCO, Life Technologies), penicillin (100 U/ml), streptomycin (100 μg/ml), and l-glutamine (4 mM) in a 5% CO_2_-humidified incubator. Experiments were performed at passage 5 or 6. A minimum of three nonasthmatic lines and three asthmatic primary cultures were used per experiment. Cells were growth arrested 24 h prior to experiment initiation. For studies in which the effect of inhibitors was assessed by measuring supernatant CXCL8 protein concentrations by ELISA, cells were cultured in 48-well plates. For real-time analysis of CXCL8 mRNA expression, HASM cells were cultured in 6-well plates. For isolation of genomic DNA, cells were cultured in T75 flasks and for chromatin immunoprecipitation (ChIP) assays, cells were cultured in T225-cm^2^ flasks.

#### BET inhibitor studies.

Media were replaced with serum-free media containing 0.1% DMSO, 0.1 μM/1 μM/10 μM PFI-1 (Tocris Bioscience, Bristol, UK), 0.1 μM/1 μM/10 μM I-BET (Tocris), or 0.01 μM/0.1 μM/1 μM JQ-1 (Cayman Chemical) compounds. Supernatants were collected 24 h later and assayed by ELISA.

#### Human CXCL8 ELISA.

The human CXCL8 ELISA (R&D Systems) was performed according to the manufacturer's protocols. CXCL8 concentrations were normalized to cell counts and expressed relative to the mean CXCL8 concentration in DMSO-treated samples in cells from nonasthmatic donors. To compare the extent of inhibition between cells from asthmatic and nonasthmatic donors we also expressed data relative to each donor cell's DMSO control.

#### RNA expression.

Media were replaced with serum-free media containing 0.1% DMSO, 10 μM PFI-1, 1 μM I-BET, or 1 μM JQ-1 compounds; 2 h later cells were lysed for RNA extraction.

#### RNA isolation and RT-PCR.

Total RNA was isolated, reverse transcribed, and subjected to real-time PCR as described previously (11). Human CXCL8 primers sequences were forward 5′-ATGACTTCCAAGCTGGCCGTGGCT-3′ and reverse 5′-TCTCAGCCCTCTTCAAAAACTTCTC-3′. β_2_-Microglobulin (β_2_M) was used as a housekeeping gene (forward 5′-AATCCAAATGCGGCATCT-3′, reverse 5′- GAGTATGCCTGCCGTGTG-3′). Expression was expressed by the ΔΔCt method relative to β_2_M Ct and within donor cell line DMSO control CXCL8/β_2_M ΔCt.

#### Cell viability.

The toxicity of all chemicals and vehicles was determined by MTT assay. At the end of the experiment culture media were removed and replaced with 250 μl of serum-free media containing 1 mg/ml thiazolyl blue, 3-(4,5-dimethylthiazol-2-yl)-2,5-diphenyltetrazolium bromide (MTT) (Sigma), and then incubated for 20 min at 37°C. This medium was removed, and the plates were dried overnight; 250 μl of dimethyl sulfoxide (DMSO) was then added to dissolve the blue-colored tetrazolium. The absorbance was read at 550 nm in a FLUOstar Omega microplate reader (BMG Labtech, Aylesbury, UK). Viability was set as 100% in control cells.

#### Pyrosequencing.

DNA was extracted by use of the QIAamp DNA Mini kit (Qiagen), and 2 μg of genomic DNA was bisulfite converted by using the EpiTect Bisulfite kit (Qiagen). Eluted DNA was used as a PCR template. Primers for PCR amplification and sequencing were designed [PyroMark Assay Design 2.0 software (Qiagen)] and are given in Table 1 and indicated in Fig. 2*A*. PCRs were performed with the HotStarTaq DNA polymerase PCR kit (Qiagen) under the following conditions: 95°C 5 min; 45 cycles of 94°C 30 s; 56°C 30 s; 72°C 30 s; finally 72°C 10 min, except the PCR primers for CpGs 7 and 8, which required an annealing temperature of 52.6°C. Amplification success was assessed by agarose gel electrophoresis and the resulting products were pyrosequenced with the Pyromark Q24 System (Qiagen).

**Table 1.** Pyrosequencing primers

CpG Site	PCR Forward Primer (5′-3′)	PCR Reverse Primer (5′-3′)	Sequencing Primer (5′-3′)
1	TGTTTATAGTGTGGGTAAATTTATTGT	ATCCTAAAAAAAAAAATCCAAAACCT (Bio)	TGGGTAAATTTATTGTTTTGT
2	as above	as above	ATAAATTATGTATTTGTTTAGAAG
3	GTGGAGTTTTAGTATTTTAAATGTATAT (Bio)	ATCACACTTCCTATTTATTCCTTATCA	ACTTCCTATTTATTCCTTATCAA
4	as above	as above	as above
5	TTGAGGGGATGGGTTATTAGTT	ACTTATACACCCTCATCTTTTCAT (Bio)	GGATGGGTTATTAGTTGTA
6	GTGTATAAGTTTTTTAGTAGGGTGATG	AATCAAAAAAACTACCAAAAAAACC (Bio)	AGGGTGATGATATAAAAAGT
7	as above	as above	AGGATAAGAGTTAGGAAGA
8	as above	as above	ATTGTGTGTAAATATGATTTTTAA

#### Chromatin immunoprecipitation.

ChIP was performed by using the ChIP-IT Express kit (Active Motif) following the manufacturer's protocol. Chromatin was fragmented by using the Q800R Sonicator (Active Motif). Antibody amounts and product codes are stated in Table 2. Input and IgG/whole serum controls were performed in parallel. Input DNA was phenol/chloroform extracted before being used in PCR. Products were amplified by quantitative real-time PCR as described previously (29). Association is expressed by the ΔΔCt method relative to Input Ct and either the mean nonasthmatic input/IP ΔCt or within-donor cell line DMSO control input/IP ΔCt for I-BET inhibitor studies.

**Table 2.** ChIP antibodies and amounts used in IPs

Antibody Target	Manufacturer	Product Code	Amount in IP
H3K9me3	Millipore	17-625	4 μg
H4K4me3	Active Motif	39159	4 μl (whole serum)
Pan H4Ac	Millipore	06-598	4 μg
Pan H3Ac	Millipore	06-599	4 μg
H3K9Ac	Active Motif	39137	10 μl (whole serum)
H3K14Ac	Active Motif	39599	10 μl (whole serum)
H3K27Ac	Active Motif	39135	4 μl (whole serum)
H3K18Ac	Active Motif	39693	4 μl (whole serum)
p300	Santa Cruz	Sc-9001X	5 μg
P/CAF	Santa Cruz	Sc-6300 X	5 μg
BRD2	Santa Cruz	Sc-46805	3 μg
BRD3	Santa cruz	Sc-99192	3 μg
BRD4	Bethyl Labs	A301-985A50	5 μg
p65	Santa Cruz	Sc-372X	5 μg
CEBP	Santa Cruz	Sc-150X	5 μg
RNA polymerase II	Santa Cruz	Sc-9001	5 μg

IPs, immunoprecipitations; CEBP, C/EBPβ.

#### Statistical analysis.

Data are expressed as means ± SE of *n* determinants. *N* is stated in figure legends. *N* refers to the number of cell donors used per experiment. Statistical analyses were performed with GraphPad Prism Software (version 6). Unpaired two-tailed Student's *t*-tests were used to determine significant differences; a *P* < 0.05 was considered significant.

## RESULTS

### 

#### Increased CXCL8 expression from ASM cells from asthmatic individuals is associated with altered histone acetylation.

**Fig. 1.**
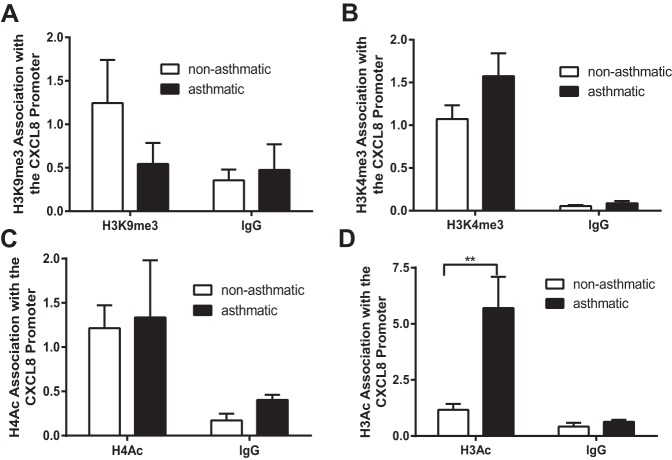
CXCL8 hypersecretion is associated with altered histone acetylation. Histone H3 lysine 9 trimethylation (H3K9me3) (*A*), histone H3 lysine 4 trimethylation (H3K4me3) (*B*), pan-acetylated histone H4 (H4Ac) (*C*), and pan-acetylated histone H3 (H3Ac) (*D*) association with the CXCL8 promoter was assessed by chromatin immunoprecipitation (ChIP). Binding was measured in confluent, serum-deprived airway smooth muscle (ASM) cells from asthmatic and nonasthmatic donors. IgG-negative controls are shown. Data are expressed as fold change relative to the mean nonasthmatic IP value. **P < 0.01 comparing asthmatic with nonasthmatic. *A*, *B*, and *D*: *n* = 3 nonasthmatic and 3 asthmatic donor ASM lines. *C*: *n* = 3 nonasthmatic and 5 asthmatic donor ASM lines.

We first investigated differences in histone modifications at the CXCL8 promoter in ASM cells from nonasthmatic vs. asthmatic individuals. Since the CXCL8 promoter has been shown previously to be regulated by histone acetylation and methylation of H3 lysine 4 and H3 lysine 9 (5), we measured the levels of these modifications at the CXCL8 promoter using ChIP and primers that amplify the region −121 to +67 bp relative to the transcription start site (29). Although histone acetylation is generally associated with active transcription, the role of histone methylation is more complex and depends on the position and extent (mono, bi, tri) of the methylation. Di- or trimethylation of lysine 9 on histone H3 (H3K9me2/3) is associated with transcription repression and heterochromatin formation (34), whereas tri- and dimethylation of lysine 4 on histone H3 (H3K4me2/3) are found at actively transcribing genes (34). Although we saw a reduced level of H3K9me3 associated with the CXCL8 promoter in cells isolated from asthmatic individuals compared with those from nonasthmatic individuals (Fig. 1*A*) and an increased association of H3K4me3 with the CXCL8 promoter in ASM cells isolated from asthmatic individuals (Fig. 1*B*), neither reached statistical significance and they were not investigated further. Subsequently we investigated histone H3 and H4 acetylation. Interestingly, although there was no difference in the levels of histone H4 acetylation at the CXCL8 promoter in cells from asthmatic individuals compared with nonasthmatic individuals (Fig. 1*C*), histone H3 acetylation at the CXCL8 promoter was increased in cells from asthmatic individuals (Fig. 1*D*) and we went on to investigate this further.

#### ASM cell CXCL8 hypersecretion from asthmatic individuals is not associated with differences in CXCL8 DNA methylation.

**Fig. 2.**
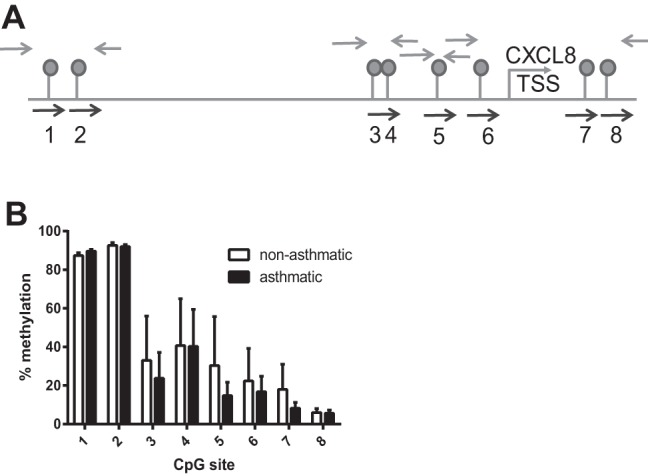
CXCL8 hypersecretion is not associated with differences in DNA methylation. Genomic DNA was isolated from ASM cells from 3 asthmatic and 3 nonasthmatic individuals and bisulfite converted. Regions of DNA containing CpG sites were PCR amplified and pyrosequenced. *A*: schematic showing the position of the 8 CXCL8 CpG sites, the PCR primers (gray arrows), and sequencing primers (black arrows). *B*: percent methylation of the individual CpG sites in ASM cells from both asthmatic and nonasthmatic individuals.

The CXCL8 gene sequence contains eight CpG sites within the region 1,500 bp upstream and 150 bp downstream of the transcription start site (Fig. 2*A*). We assessed the percent methylation at each CpG site in ASM cells isolated from asthmatic individuals and nonasthmatic controls by quantitative pyrosequencing. CpG site methylation varied across the DNA (Fig. 2*B*). Methylation was highest at the CpGs located distally upstream of the transcription start site and decreased to a minimum level at CpG8, located downstream of the transcription start site. However, we observed no difference in CXCL8 CpG methylation between cells isolated from asthmatic individuals vs. nonasthmatic individuals, suggesting that DNA methylation does not play in a role CXCL8 hypersecretion from asthmatic ASM cells.

#### Increased acetylation at the asthmatic CXCL8 promoter is specific to histone H3 lysine 18 acetylation and is associated with increased histone acetyltransferase binding.

**Fig. 3.**
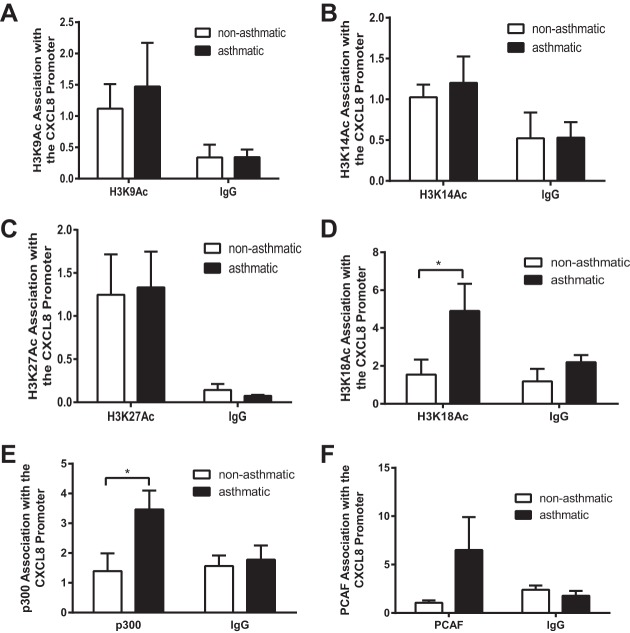
Increased CXCL8 promoter histone acetylation is specific to histone H3 lysine 18 acetylation and is associated with increased acetyltransferase binding. Histone H3 lysine 9 acetylation (H3K9Ac) (*A*), histone H3 lysine 14 acetylation (H3K14Ac) (*B*), histone H3 lysine 27 acetylation (H3K27Ac) (*C*), histone H3 lysine 18 acetylation (H3K18Ac) (*D*), p300 (*E*), and p300/CBP-associated factor (PCAF) association with the CXCL8 promoter (*F*) were assessed by ChIP. Binding was measured in confluent, serum-deprived ASM cells from asthmatic and nonasthmatic donors. IgG-negative controls are shown. Data are expressed as fold change relative to the mean nonasthmatic IP value. *P < 0.05 comparing asthmatic with nonasthmatic. *A*, *B*, *C*, *E*, and *F*: *n* = 3 nonasthmatic and 3 asthmatic donor ASM lines. *D*: *n* = 4 nonasthmatic and 3 asthmatic donor ASM lines.

To understand in greater the detail the histone H3 acetylation landscape at the CXCL8 promoter in ASM cells from asthmatic and nonasthmatic donors, we performed ChIP using antibodies for four specific histone acetylation sites: lysine 9 (H3K9Ac), lysine 14 (H3K14Ac), lysine 27 (H3K27Ac), and lysine 18 (H3K18Ac). Although we found no differential in acetylation between the asthmatic and nonasthmatic cells at sites K9, K14, and K27 (Fig. 3, *A–C*) we found that H3K18Ac was barely detectable over the IgG control in ASM cells from nonasthmatic donors but was increased in the cells isolated from asthmatic individuals (Fig. 3*D*).

To understand why H3K18Ac was increased we investigated the binding of HATs, the enzymes responsible for depositing acetyl groups on histone tail lysine residues, to CXCL8 promoter. There are ∼30 known HATs in humans that are grouped into five families based on the structural and functional similarity of their catalytic domains. Here we focused on the HATs p300 (28) and P300/CBP-associated factor (PCAF) (1) because they are known to acetylate H3K18. As with H3K18Ac we observed little signal for either p300 (Fig. 3*E*) or PCAF (Fig. 3*F*) over the IgG control in nonasthmatic ASM cells but observed increased binding in asthmatic ASM cells, with p300 reaching statistical significance. This suggests that aberrant H3K18Ac at the CXCL8 promoter in ASM cells from asthmatic donors is caused by inappropriate recruitment of HATs to the CXCL8 promoter.

#### The histone acetylation reader proteins, Brd3 and Brd4, are present at the CXCL8 promoter and BET inhibitors reduce CXCL8 secretion from ASM cells.

**Fig. 4.**
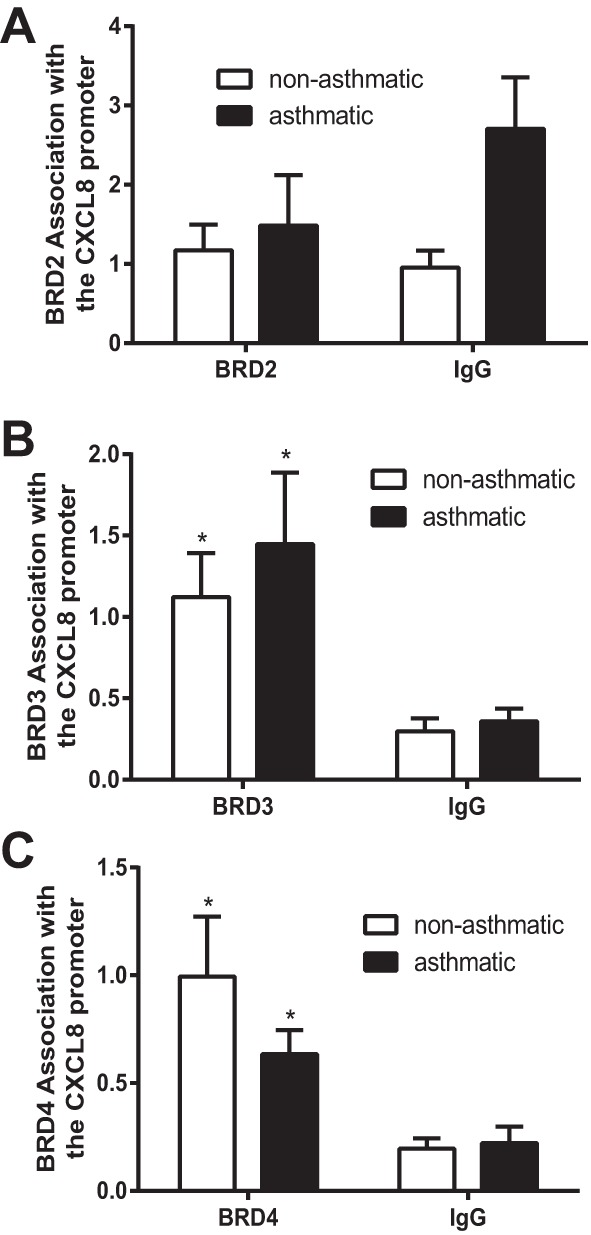
Brd3 and Brd4 are present at the CXCL8 promoter. Brd2 (*A*), Brd3 (*B*), and Brd4 (*C*) association with the CXCL8 promoter was assessed by ChIP. Binding was measured in confluent, serum-deprived ASM cells from asthmatic and nonasthmatic donors. IgG-negative controls are shown. Data are expressed as fold change relative to the mean nonasthmatic IP value. *P < 0.05 comparing target IP to IgG control. *A* and *B*: *n* = 5 nonasthmatic and 5 asthmatic donor ASM lines. *C*: *n* = 4 nonasthmatic and 4 asthmatic donor ASM lines.

Bromodomain (BRD)-containing proteins are a class of histone modification reader proteins that specifically recognize acetylated lysine residues. They play crucial roles in transcriptional regulation by acting as scaffolds for macromolecular complexes that alter chromatin accessibility to transcription factors and RNA polymerases. The BET proteins, Brd2, Brd3, Brd4, and BrdT, are one family of BRD-containing proteins. Importantly, the first selective nanomolar inhibitors for these proteins have been identified and have shown translatable promise in models of midline carcinoma (21) and lipopolysaccharide-induced endotoxic shock and bacteria-induced sepsis (40). This is in contrast to direct inhibitors of histone acetylation, which have struggled in drug development owing to lack of selectivity and to pleotropic effects. Because of the fact that histone acetylation is present and dysregulated at the CXCL8 promoter, we aimed to identify whether BET family proteins were bound to the CXCL8 promoter. We performed ChIP for Brd2, 3, and 4. BrdT was not investigated because it has testis-specific expression. ChIP for BRD2 showed no enrichment for BRD2 binding over IgG control (Fig. 4*A*), suggesting BRD2 is not associated with the CXCL8 promoter. However, both BRD3 (Fig. 4*B*) and BRD4 (Fig. 4*C*) were associated with the CXCL8 promoter (significant difference between target IP and IgG). No differences in BRD protein binding were seen between cells isolated from asthmatic donors and those from nonasthmatic donors.

**Fig. 5.**
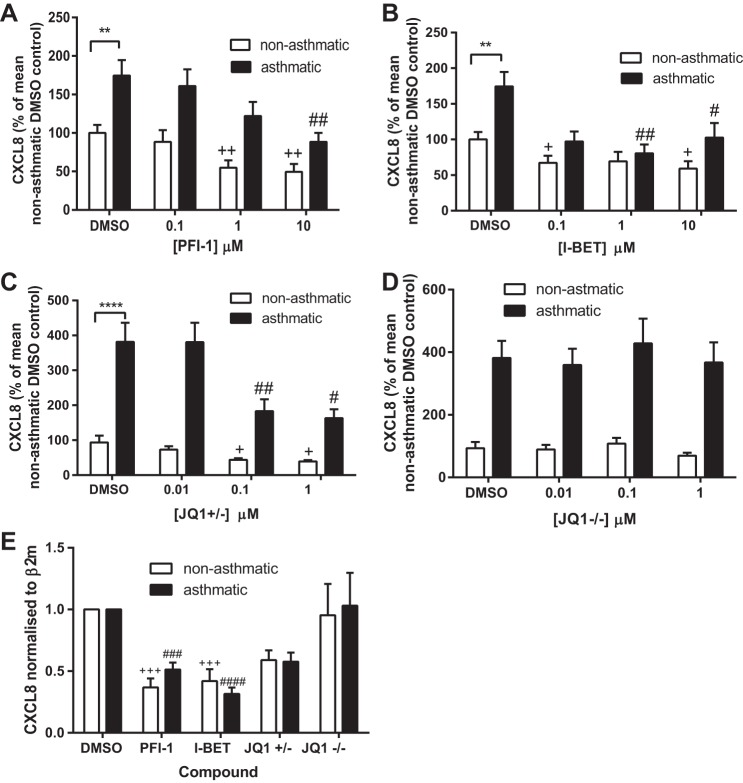
Bromodomain and extraterminal (BET) inhibitors reduce CXCL8 secretion and expression from ASM cells. CXCL8 protein levels following incubation with PFI-1 (*A*), I-BET (*B*), JQ+/− (*C*), and JQ−/− (*D*). Supernatants were collected from confluent, serum-deprived ASM cells following 24 h incubation with the stated concentration of compound. **P < 0.01, ****P < 0.0001 comparing asthmatic with nonasthmatic DMSO control. +*P* < 0.05, ++*P* < 0.01 compared with nonasthmatic DMSO control. #*P* < 0.05, ##*P* < 0.01 compared with asthmatic DMSO control. *E*: CXCL8 mRNA levels as determined by real-time PCR following 2 h incubation with 10 μM PFI, 1 μM I-BET, 1 μM JQ+/−, and 1 μM JQ−/−. Data are expressed relative to each donor line's DMSO control. β_2_-Microglobulin (β_2_M) was used as a housekeeping gene. +++*P* < 0.001, compared with nonasthmatic DMSO control. ###*P* < 0.001, ####*P* < 0.0001 compared with asthmatic DMSO control. *A* and *B*: *n* = 3 nonasthmatic and 3 asthmatic donors. *C* and *D*: *n* = 5 nonasthmatic and 4 asthmatic donors. *E*: *n* = 4 nonasthmatic and 4 asthmatic donors.

**Fig. 6.**
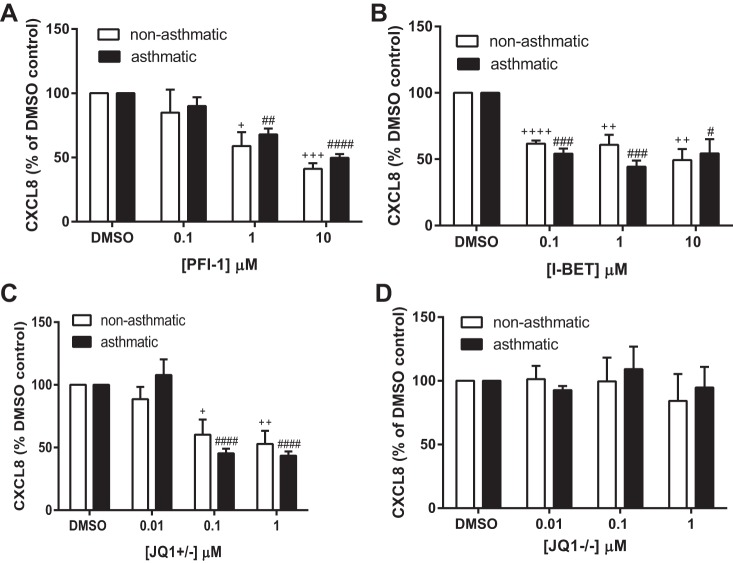
There is no difference in the BET inhibitory effect between ASM cells from asthmatic and nonasthmatic donors. CXCL8 protein levels following incubation with PFI-1 (*A*), I-BET (*B*), JQ+/− (*C*), and JQ−/− (*D*). Supernatants were collected from confluent, serum-deprived ASM cells following 24 h incubation with the stated concentration of compound. +*P* < 0.05, ++*P* < 0.01, +++*P* < 0.001, ++++*P* < 0.0001 compared with nonasthmatic DMSO control. #*P* < 0.05, ##*P* < 0.001, ###*P* < 0.001, ####*P* < 0.0001 compared with asthmatic DMSO control. *A* and *B*: *n* = 3 nonasthmatic and 3 asthmatic donors. *C* and *D*: *n* = 5 nonasthmatic and 4 asthmatic donors.

**Fig. 7.**
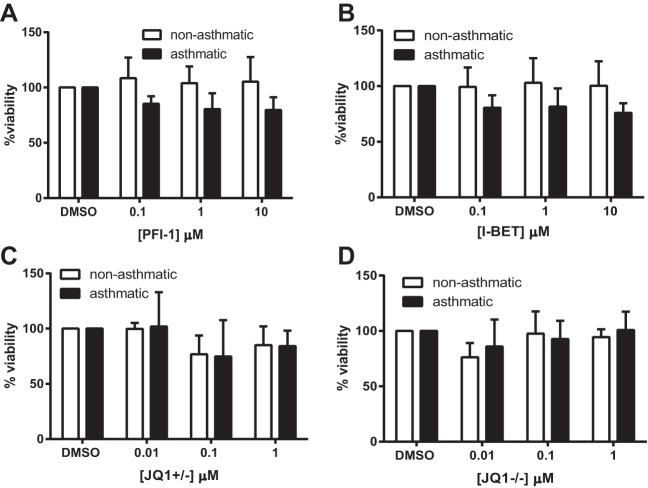
BET inhibitors are not toxic to ASM cells. Percent cell viability following incubation with PFI-1 (*A*), I-BET (*B*), JQ+/− (*C*), and JQ−/− (*D*); 250 μl of 1 mg/ml MTT solution was added to rinsed ASM cells following removal of the supernatants. Cells were incubated for 20 min at 37°C, and the MTT was removed and dried overnight. The 250 μl DMSO was used to resuspend the MTT and the absorbance at 550 nM was read. Data are expressed as percentages relative to DMSO controls and are derived from the same *n* as matched experiments in Fig. 5, *A–D*.

To determine whether BET proteins were regulating CXCL8 expression we measured the effect of three different BET protein inhibitors on CXCL8 protein secretion and mRNA expression in ASM cells from both asthmatic and nonasthmatic donors. Three structurally different compounds we used were the potent and highly selective dihydroquinazoline-2-one inhibitor PFI-1 (44), I-BET (40), and the thienodiazepime JQ1. The enantiomerically pure (+)-JQ1 inhibits BET proteins whereas the (−)-JQ1 stereoisomer has no effect and can be used as a negative control compound (21). Here cells were serum starved for 24 h, the media were replaced with fresh media containing the stated concentration of compound, and supernatants and RNA samples were collected at 24 and 2 h, respectively. As shown previously, ASM cells from asthmatic donors secreted significantly more CXCL8 than those from nonasthmatic donors (data shown are percent CXCL8 relative to the mean CXCL8 levels of nonasthmatic DMSO samples). PFI-1 (Fig. 5*A*), I-BET (Fig. 5*B*), and JQ1+/− (Fig. 5*C*) significantly reduced CXCL8 protein secretion into the cell culture supernatant. The negative control compound JQ1−/− had no effect on CXCL8 secretion (Fig. 5*D*). When data were expressed as a percentage of each donor cell's DMSO control (Fig. 6) all compounds showed a similar extent of inhibition at the highest concentration used, with the percent CXCL8 remaining ranging from 41.1% (10 μM PFI-1 with nonasthmatic cells) to 54.3% (10 μM I-BET with asthmatic cells). There was no difference in the extent of inhibition between ASM cells isolated from asthmatic donors vs. those from nonasthmatic donors. To ensure that inhibition was not accounted for by cell toxicity, MTT assays were performed. As shown in Fig. 7 none of the inhibitors or the JQ−/− control compound caused any cell toxicity. CXCL8 mRNA expression (Fig. 5*E*) was significantly inhibited by 10 μM PFI-1 and 1 μM I-BET in ASM cells from both asthmatic and nonasthmatic individuals; 1 μM JQ1+/− showed a decrease in CXCL8 mRNA levels compared with 1 μM JQ1−/− in both ASM cells from asthmatic and nonasthmatic donors. These data suggest that BET proteins are involved in the transcriptional regulation of CXCL8. This may offer a novel, indirect, and potentially translatable approach to modulating aberrant histone acetylation-dependent gene expression in asthma, by targeting the reader BET proteins rather than the writer HATs directly.

#### BET protein inhibitors mediate their action via disruption of BRD4 and RNA polymerase II, but not transcription factor, association with the CXCL8 promoter.

**Fig. 8.**
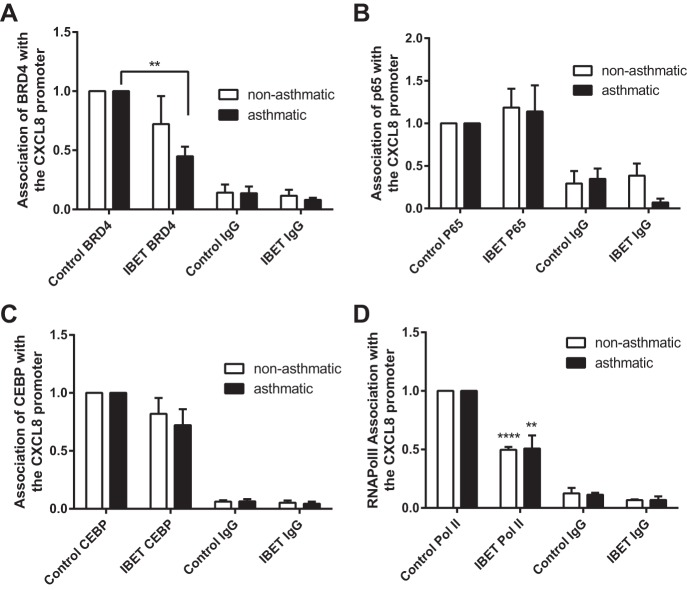
BET inhibitors mediate their effects via disruption of Brd4 and RNA polymerase II association with the CXCL8 promoter. Changes in Brd4 (*A*), p65 (*B*), C/EBPβ (CEBP; *C*), and RNA polymerase II (RNAPolII; *D*) association with the CXCL8 promoter following 30-min incubation with 1 μM I-BET was assessed by ChIP. Binding was measured in confluent, serum-deprived ASM cells from asthmatic and nonasthmatic donors. IgG-negative controls are shown. Data are expressed as fold change relative to within donor control IP value. **P < 0.01, ****P < 0.0001 comparing I-BET with DMSO control. *A* and *B*: *n* = 4 nonasthmatic and 4 asthmatic donor ASM lines. *C* and *D*: *n* = 3 nonasthmatic and 4 asthmatic donor ASM lines.

Finally we wanted to establish the molecular mechanism of CXCL8 inhibition by BET inhibitors. The three BET inhibitors used are known to act by mimicking acetylated histones and preventing Brd protein chromatin association. As such, we performed ChIP for Brd4 and Brd3 on chromatin from cells cultured with or without the BET inhibitors PFI-1 and I-BET (10 μM and 1 μM, respectively). Preliminary single cell line time course experiments (data not shown) showed that BRD3 binding was not affected by either 1 μM I-BET or 10 μM PFI-1 and that BRD4 binding to the CXCL8 promoter was more efficiently inhibited by I-BET than PFI-1. BRD4 dissociation had occurred within 30 min of BET protein inhibitor incubation (data not shown). A subsequent ChIP experiment showed that I-BET significantly inhibited BRD4 binding to the CXCL8 promoter in ASM cells from asthmatic donors and reduced binding in cells from nonasthmatic donors (Fig. 8*A*). We have shown previously that NF-κB p65 is present and active in the nucleus of unstimulated ASM from asthmatic and nonasthmatic individuals and that hypersecretion of CXCL8 from ASM cells from asthmatic donors is associated with increased binding of the transcription factors NF-κB p65 and C/EBPβ (29). Inhibition of hydrogen peroxide and interleukin-1β-stimulated CXCL8 secretion from immortalized human bronchial epithelial cells by JQ1+/− is mediated via inhibition of p65 binding to the CXCL8 promoter (31). Hence we investigated whether in the present context the action of I-BET was mediated via modulation of p65 and C/EBPβ binding to the CXCL8 promoter. As shown in Fig. 8, *B* and *C*, respectively, whereas p65 and C/EBPβ are present at the CXCL8 promoter, I-BET had no effect on p65 binding and little effect on C/EBPβ binding. Finally, because RNA polymerase II is required for mRNA transcription and we have previously shown increased RNA polymerase II association with the CXCL8 promoter in asthmatic ASM cells, we investigated the effect of BET protein inhibition on RNA polymerase II association. Figure 8*D* shows that 1 μM I-BET significantly reduced the levels of RNA polymerase II associated with the CXCL8 promoter in ASM cells from both asthmatic and nonasthmatic individuals. These data suggest that BET protein inhibitors mediate their effects at the CXCL8 promoter in ASM cells via disruption of BRD4 and RNA polymerase II while leaving the measured transcription factor complex in place.

## DISCUSSION

The main finding in this study is that histone acetylation is dysregulated at the CXCL8 promoter in ASM cells from asthmatic donors. Furthermore, we show that the histone acetylation binding BET proteins are critical to CXCL8 expression and that BET protein inhibitors offer a novel therapeutic option for modulating CXCL8 in asthma and potentially other neutrophilic diseases.

We chose to study CXCL8 because it is a key chemoattractant for neutrophils and neutrophilic inflammation is a feature of steroid-resistant asthma, which is difficult to treat. Studies in humans with asthma and mouse asthma models show that CXCL8 blocking strategies are beneficial. The selective CXCR2 receptor antagonist SCH527123 reduced sputum neutrophil counts and mild exacerbations in severe asthma patients (39). Furthermore, blockade of the murine CXCL8 receptors CXCR1 and CXCR2 reduced neutrophilic airway inflammation in mouse models of various pulmonary conditions including asthma (23). We studied CXCL8 expression regulation in cultured ASM cells from asthmatic and nonasthmatic donors because we have previously shown that cells from asthmatic donors hypersecrete CXCL8 (29). This change in expression is stable with repeated passage in culture, suggesting a fundamental change in cell phenotype. Furthermore, changes in cytokine expression are seen in intact human asthma tissue (45).

We found that the most striking observation at the CXCL8 promoter in cells from asthmatic donors was a large increase in acetylated histone H3. We previously described dysregulated histone lysine methylation regulating aberrant VEGF expression in ASM cells from asthmatic patients (11). Specifically, a lack of the repressive H3K9me3 mark and an increase in the active H3K4me3 mark was observed at the VEGF promoter in cells from asthmatic donors. Although we did see some reduction in H3K9me3 and some increase in H3K4me3 at the CXCL8 promoter in ASM cells from asthmatic donors, these histone modifications were not statistically significant. Similarly in our previous studies of the VEGF promoter no changes in histone H3 acetylation were seen. These data suggest that where there is a lack of difference between our cells from asthmatic and nonasthmatic donors it is due to different underlying molecular mechanisms at the promoters rather than technical experiential issues. Acetylation regulates gene expression via two primary methods: alteration of the nucleosome confirmation from a closed to an open state, allowing access for the transcriptional machinery, and the generation of binding motifs on the nucleosome surface for reader/effector proteins that assist in recruiting the molecular complexes required for active transcription (10). Previous studies have shown that CXCL8 expression is regulated by either histone H3 or H4 histone acetylation in several cell types and in response to a range of stimuli (3, 8), and we have shown that TNF-α induction of CXCL8 expression in ASM cells is associated with histone H4 acetylation (41). However, to our knowledge regulation of CXCL8 by histone H3 acetylation has not previously been reported in ASM cells. Furthermore, although most previous publications have measured pan-histone acetylation, we investigated four specific histone H3 acetylation sites and identified dysfunction in a single site, H3K18. Few studies have aimed to identify the individual roles of specific histone lysine residue acetylation. Those studies that have are mainly in cancers. In *Saccharomyces cerevisiae* it was proposed that lysines 4, 9, 14, 18, 23, and 27 on histone H3 have redundant functions and act as a group to mark euchromatin; however, the investigators did not include mutation analysis of K18 alone in their study (37). H3K18 deacetylation is required for host responses to bacterial infection (18) and H3K18 hypoacetylation is linked to maintenance of a malignant phenotype (7) and poor prognosis in prostate cancer (49) and pancreatic adenocarcinoma (36), suggesting that acetylation of lysine 18 has a specific cellular role. It would be of interest in future studies to assess whether increased H3K18Ac is more widespread in ASM cells from asthma subjects (and whole lung tissue incorporating the many cell types involved in asthma pathogenesis) or whether it is specific to CXCL8. The increased H3K18 acetylation was associated with a concomitant increase in association of the acetylation writer protein, HAT p300. We also observed a trend toward increased PCAF binding. Both p300 and PCAF can acetylate multiple histone lysine residues on both histone H3 and H4 (26, 48) and it is unclear at present what regulates the sites they acetylate under any given condition. Speculatively the presence of neighboring or associated histone modifications may play a role. We attempted to assess this here but experienced technical barriers. Because the lack of specificity and toxicity of available HAT inhibitors makes them unsuitable to unravel the molecular function of H3K18ac in whole cell systems we performed siRNA studies to knock down p300 and PCAF (data not shown). Unfortunately this approach induced cell toxicity, likely reflecting the fundamental roles of these proteins in regulating diverse cellular functions including cell growth, differentiation, and apoptosis.

It would be of further future interest to understand the mechanisms driving altered HAT association with the CXCL8 promoter. Changes in the epigenetic profiles of airway structural cells are likely initiated by insults from inhaled stimuli, including allergens, respiratory viruses, and air pollutants. Little is known about the direct effect of allergens on epigenetic mechanisms including HAT recruitment. However, in response to allergens, T helper (Th) lymphocytes are differentiated into Th2 cells, and Th2 differentiation is accompanied by alterations in the acetylation state of histones at cytokine gene loci including the allergic inflammatory cytokine interleukin-4 (19, 25) and interferon gamma (19), suggesting that HAT activation occurs in response to allergen exposure (27). A major air pollutant, diesel exhaust, regulates cyclooxygenase 2 (COX-2) expression in airway epithelial cells via increased recruitment of p300 to the COX-2 promoter (9). Although little is known about respiratory virus effects on HATs in the lung, viruses are well known to interact with HATs in cancer. The HPV 6, 16, and 18 E7 proteins of a high-risk human papillomavirus (HPV) involved in cervical cancer directly interact with PCAF (4) and the cellular protein AMF-1 (Gps2) positively modulates gene expression by the papillomavirus E2 protein via direct interaction with p300 (43). Taking this evidence into account, we speculate that exposure to inhaled environmental exposures induces stable alterations in the recruitment of epigenetic writer proteins, including HATs, to specific DNA regions in airway structural cells including ASMs that affect specific gene expression and cell function and contribute to asthma pathogenesis.

In addition to assessing histone changes we investigated the DNA methylation status of CpG sites within the CXCL8 promoter. The majority of DNA methylation studies are performed at genes that contain large regions rich in CpG sites (CpG islands) as hypermethylation of cytosines within these regions silences gene transcription. Although the CXCL8 promoter does not contain a CpG island and is therefore an unlikely candidate for DNA methylation analysis, dysregulated methylation of individual CpG sites within the CXCL8 promoter has been described in non-small cell lung cancer (51) and colorectal adenocarcinoma (16) and associated with altered gene expression. We found no differences in CXCL8 promoter DNA methylation between ASM cells from asthmatic and nonasthmatic donors. Our study is however, the first to quantitatively assess methylation of all eight CpGs within the 1,650-bp region of the CXCL8 transcription start site. We found that there was a decrease in CpG methylation as proximity to the transcription start site was reduced consistent with a recent genomewide study of DNA methylation in 17 somatic tissues that showed decreasing methylation the closer to the TSS at non-CpG island DNA regions (35).

No highly specific HAT inhibitors have been developed, and we postulated that targeting “reader” proteins might prove a better therapeutic strategy in our cells. Reader proteins recognize histone modification marks and either play a direct role in transcriptional regulation or recruit proteins/complexes that regulate transcription. In contrast to HATs, BET proteins have been shown to be targetable by selective inhibitors that have a good therapeutic profile. We found that two BET proteins, Brd3 and Brd4, were present at the CXCL8 promoter in ASM cells from both asthmatic and nonasthmatic donors and that three different BET protein inhibitors, PFI-1, I-BET, and JQ+/−, reduced CXCL8 protein secretion and mRNA levels. There were no differences in the levels of Brd3 or Brd4 recruitment to the CXCL8 promoter in cells from asthmatic vs. nonasthmatic donors. This is not necessarily surprising because we observe very specific differential histone H3 lysine 18 acetylation at the asthmatic CXCL8 promoter and the Brd proteins are likely also associated with other acetylated residues at the CXCL8 promoter. What is important to the present study is that inhibition of these BET proteins normalized the levels of CXCL8 secretion from ASM cells from asthmatic patients to those secreted from ASM cells from nonasthmatic individuals. JQ+/− has previously been shown to inhibit hydrogen peroxide- and interleukin-1β-induced CXCL8 secretion from immortalized airway epithelial cells (31), but ours is the first report of its efficacy in primary ASM cells and its ability to normalize aberrant cytokine secretion in cells from asthmatic donors. Our results differ from those of Khan et al. (31), who found the BET inhibitor effect in immortalized airway epithelial cells to be mediated via disruption of Brd4 and p65 binding to the CXCL8 promoter whereas we observed no effect on p65 or C/EBPβ transcription factor binding but rather a reduction in Brd4 and RNA polymerase II association with the CXCL8 promoter. Interestingly, the Khan study also showed that whereas reduction of Brd4 protein levels mimicked the effects of the pharmacological I-BET inhibitor, reduction of Brd2 protein levels had no such effect. Similarly, in our study we show that I-BET disrupts Brd4 association with the CXCL8 promoter but has no effect on Brd3 binding (data not shown). This suggests that these inhibitors may exhibit context specific effects. BET inhibitor compounds were not toxic to our primary ASM cells (MTT assays), adding to their potential as therapeutics.

Although our studies focused on ASM CXCL8 production in asthma, the findings may have broader applicability to other diseases driven by neutrophilic inflammation. In rheumatoid arthritis, a chronic inflammatory disease affecting the diarthrodial joints, CXCL8 mediates neutrophil recruitment (32) and BET protein inhibition reduces collagen induced arthritis in mice (38). BET inhibitors also protect against lipopolysaccharide-induced endotoxic shock and bacteria-induced sepsis (40) and cause long-lasting suppression of Th1 cell proinflammatory function (6), via regulation of inflammatory gene expression. Heart failure includes inflammatory pathway activation in its pathology (2) and plasma CXCL8 levels correlate with worsening chronic heart failure (13), and in an in vitro phenylephrine-induced model of heart failure in neonatal rat ventricular cardiomyocytes BET protein inhibition reduced expression of proinflammatory genes (2). All of these studies highlight a strong involvement of BET proteins in inflammatory disease.

**Fig. 9.**
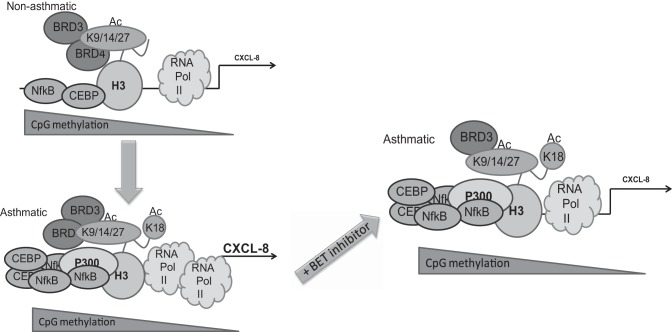
Summary schematic. Fundamental differences exist between the molecular complexes at the CXCL8 promoter in ASM cells from asthmatic and nonasthmatic donors. This is characterized by increased RNA polymerase II, C/EBPβ, and p65 binding and by aberrant presence of H3K18Ac and p300 at the asthmatic promoter. CpG methylation decreases as proximity to the transcription start site increases but is not different between ASM cells from asthmatic and nonasthmatic donors. CXCL8 transcription is dependent on the presence of histone acetylation reader proteins Brd3 and Brd4 and BET protein inhibitors can reduce asthmatic CXCL8 expression to nonasthmatic levels via disruption of Brd4 and RNA polymerase II association with the promoter.

In summary, our study strongly suggests that the chromatin/transcriptional complex at the unstimulated CXCL8 promoter is fundamentally different in ASM cells isolated from asthmatic individuals compared with nonasthmatic individuals. This is characterized by increased RNA polymerase II, C/EBPβ, and p65 binding [previously published (29)] and aberrant presence of H3K18Ac and p300 at the asthmatic promoter (Fig. 9). Furthermore, CXCL8 transcription is dependent on the presence of histone acetylation reader proteins Brd3 and Brd4, and BET protein inhibitors can modulate CXCL8 expression via disruption of Brd4 and RNA polymerase II association with the promoter. BET protein inhibitors may have therapeutic potential against steroid-resistant neutrophilic asthma and other inflammatory diseases in which neutrophilic inflammation is driven by CXCL8.

## GRANTS

This work was supported by a Wellcome Trust Programme Grant, the European Respiratory Society (ERS) and the Canadian Thoracic Surgery (CTS)/Canadian Lung Association (CLA), joint ERS/CTS Long-Term Research fellowship LTRF 2013 (R. Clifford), and a NC3Rs David Sainsbury Fellowship (A. Tatler).

## DISCLOSURES

No conflicts of interest, financial or otherwise, are declared by the author(s).

## AUTHOR CONTRIBUTIONS

R.L.C., A.E.J., A.L.T., and A.J.K. conception and design of research; R.L.C., J.K.P., A.E.J., and L.M. performed experiments; R.L.C. and A.E.J. analyzed data; R.L.C., A.E.J., and A.J.K. interpreted results of experiments; R.L.C. prepared figures; R.L.C. and A.J.K. drafted manuscript; R.L.C., A.E.J., A.L.T., C.E.B., and A.J.K. edited and revised manuscript; R.L.C., J.K.P., A.E.J., L.M., C.E.B., and A.J.K. approved final version of manuscript.
